# *In-situ* synchrotron X-ray diffraction data for the dynamic reaction processes between titanium and air under laser irradiation

**DOI:** 10.1016/j.dib.2020.105155

**Published:** 2020-01-21

**Authors:** Congyuan Zeng, Hao Wen, Hong Yao, P.T. Sprunger, S.M. Guo

**Affiliations:** a Department of Mechanical & Industrial Engineering, Louisiana State University, Baton Rouge, LA, 70803, United States; b Department of Physics & Astronomy and Center for Advanced Microstructures and Devices, LSU, Baton Rouge, LA, 70803, United States

**Keywords:** *In-situ* synchrotron X-ray diffraction, Laser irradiation, Titanium oxidation, Titanium nitridation

## Abstract

This article presents data related to the research article entitled “Diffusion of oxygen and nitrogen into titanium under laser irradiation in air” [1]. When irradiated with varying laser parameters under ambient air, titanium surfaces are observed to exhibit differing colors. To better understand this phenomenon, the dynamic reaction steps between titanium and air under laser irradiation were investigated with *in-situ* synchrotron X-ray diffraction method. With a programmed laser profile, a set of diffraction patterns were collected by a 2D detector and then analyzed with the program FIT2D. Based on the data, the detailed high-temperature reactions between titanium and air during laser irradiation were clearly revealed. The presented raw in-situ synchrotron X-ray diffraction data can be reused for the further insights of laser surface modification of titanium in air, or for discovering the optimal laser conditions for industrial decoration or medical applications of titanium.

**Table:** Specifications Table

Subject	Materials Science Surfaces, Coatings and Films
Specific subject area	Laser surface modification of titanium under air
Type of data	Image Figure
How data were acquired	*In-situ* synchrotron X-ray diffraction test Instruments: 1. Protein Crystallography synchrotron beamline (1.3GeV electron storage ring, X-ray wavelength 1.3808 Å) at the Center for Advanced Microstructures and Devices (CAMD), Baton Rouge, Louisiana, USA. 2. IPG YLS-2000 fiber laser (Continuous wave mode, Gaussian energy distribution, maximum power 2000 W). 3. PILATUS 100 K (Dectris AG, Switzerland) detector system, with the maximum framing rate of 100 Hz.
Data format	Raw TIF image
Parameters for data collection	Synchrotron X-ray wavelength: 1.3808 Å. Detector frame rate: 10 Hz. Laser power was first increased from 0 W to 200 W in 5 s with the rate of 40 W/s, then kept at 200 W for 1 s, finally decreased to 0 W in 1 s with the rate −200 W/s.
Description of data collection	The synchrotron X-ray beam was perpendicular to the laser beam direction. Prior to the test, the titanium sample surface normal was tilted around 20° to the laser beam direction for a better overlapping of the synchrotron X-ray beam spot and the laser beam spot on the sample surface. The synchrotron X-ray and the detector were simultaneously switched on around 1 s before the initiation of laser, recording the diffraction patterns of titanium in the air environment. About 1 s later, the laser was initiated and followed the laser profile aforementioned. After the laser was off, the detector and the synchrotron X-ray were kept on working for about 4 more seconds. The total data recording process was 12 s.
Data source location	Center for Advanced Microstructures and Devices (CAMD) Baton Rouge, Louisiana USA
Data accessibility	1. Raw image data are deposited at Mendeley Database with the following link: https://doi.org/10.17632/8fmsg3jnzc.1 2. Preliminarily analyzed data: With the article
Related research article	Author's name: Congyuan Zeng, Hao Wen, Boliang Zhang, P.T. Sprunger, S.M. Guo Title: Diffusion of oxygen and nitrogen into titanium under laser irradiation in air Journal: Applied Surface Science DOI: https://doi.org/10.1016/j.apsusc.2019.144578

**Table:** 

**Value of the Data** • The data in this article reveal the *in-situ* reaction steps (especially high temperature reactions) between titanium and air under laser irradiation, which help to better understand the laser surface modification process of titanium in air. • The data in this article will benefit researchers who need detailed *in-situ* reaction data on laser surface modification of titanium under air, or those who apply surface modification strategies for decoration or medical applications, or even those who investigate titanium and its alloys under laser-based additive manufacturing processes. • These data present a technique for investigating *in-situ* interactions, such as laser heating induced chemical reactions or phase changes. This technique can also be used for insights of *in-situ* interactions of other reaction systems, i.e. the sintering process of titanium and aluminium. Besides, according to the titanium-air interaction steps revealed by the data, through adjusting the laser parameters, TiN/TiO_2_ layer with varying thickness can be obtained. • In addition, these data will be beneficial for improved modeling data of the titanium-air interactions under laser irradiation.

## 1 Data

**Fig. 1:**
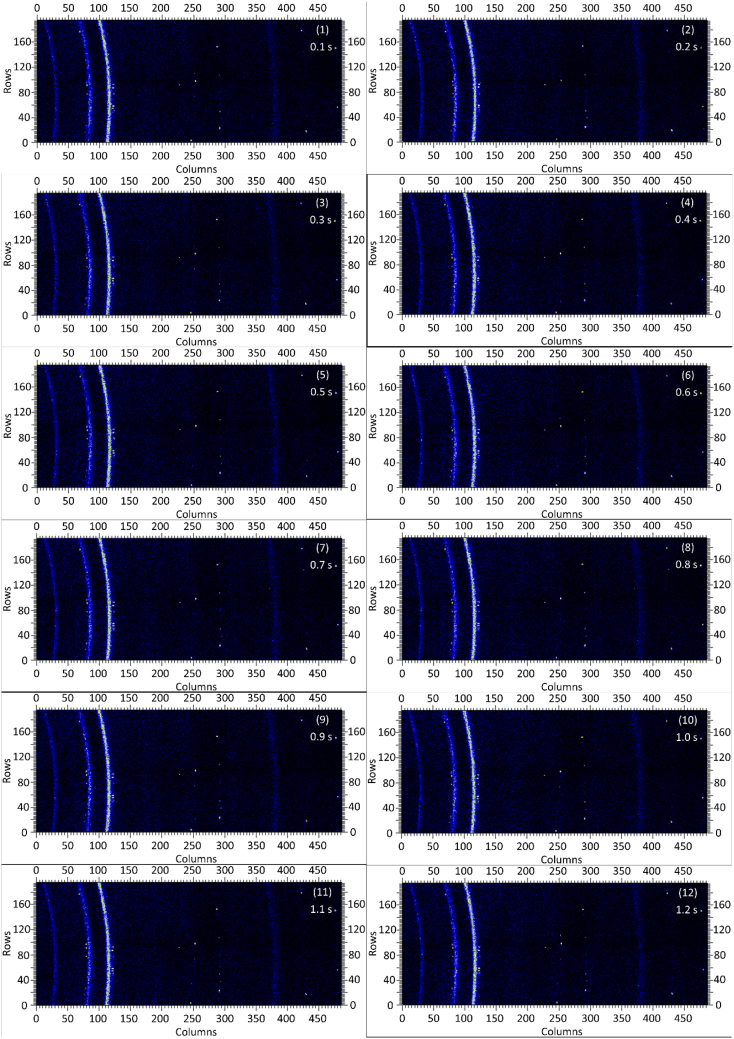

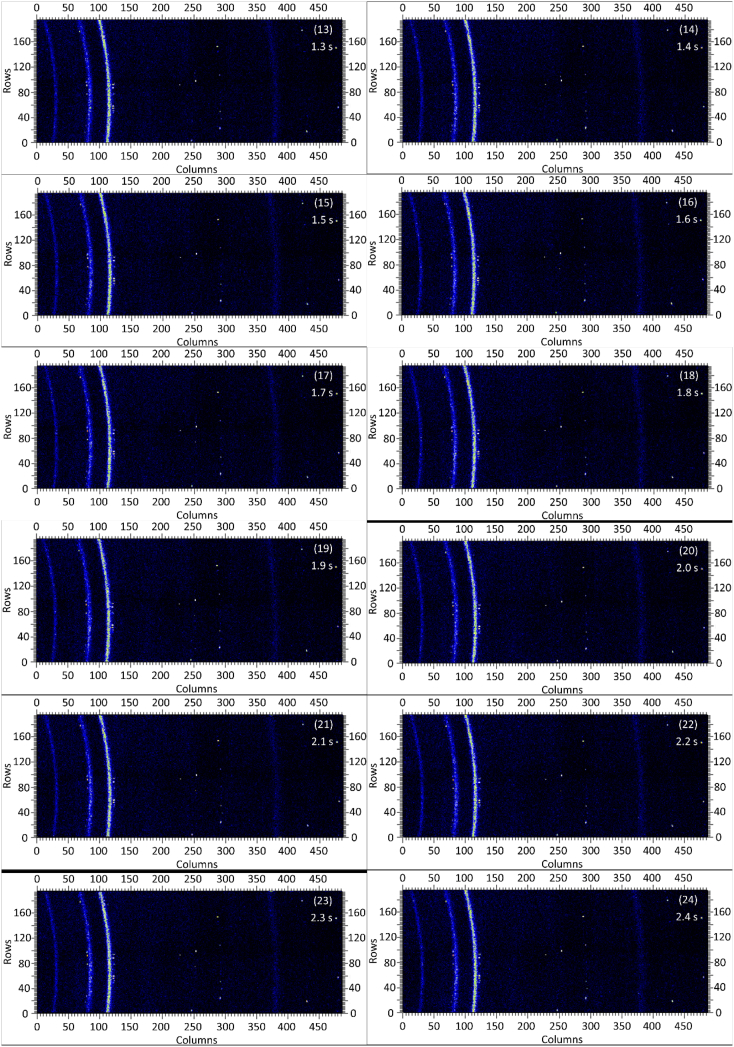

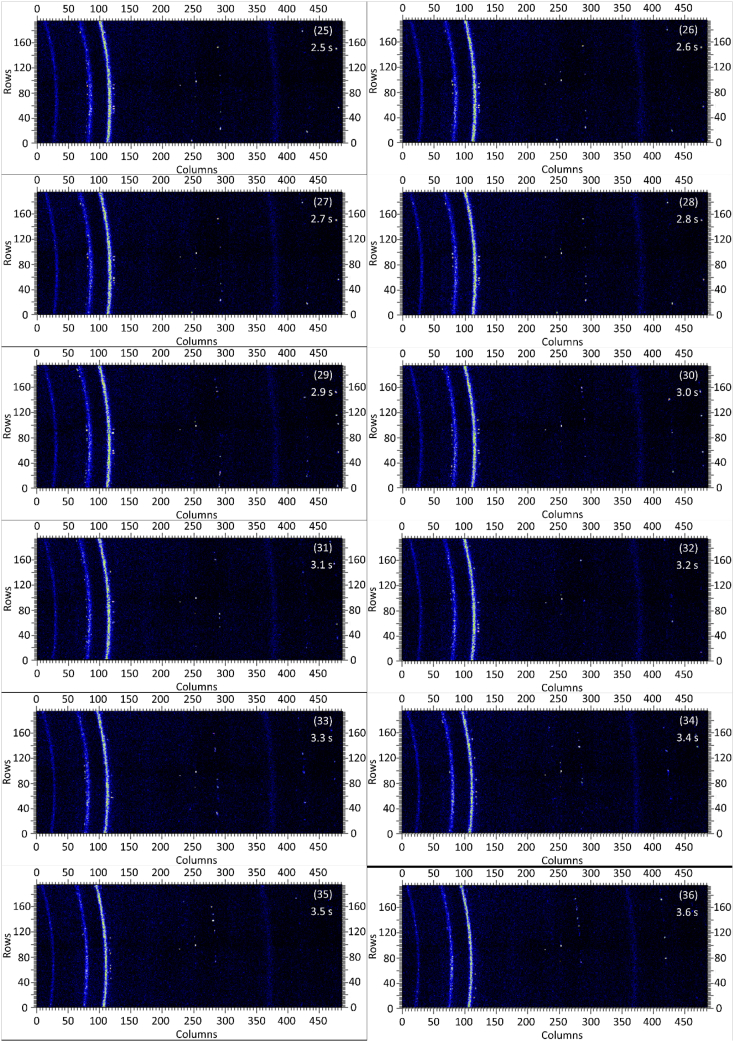

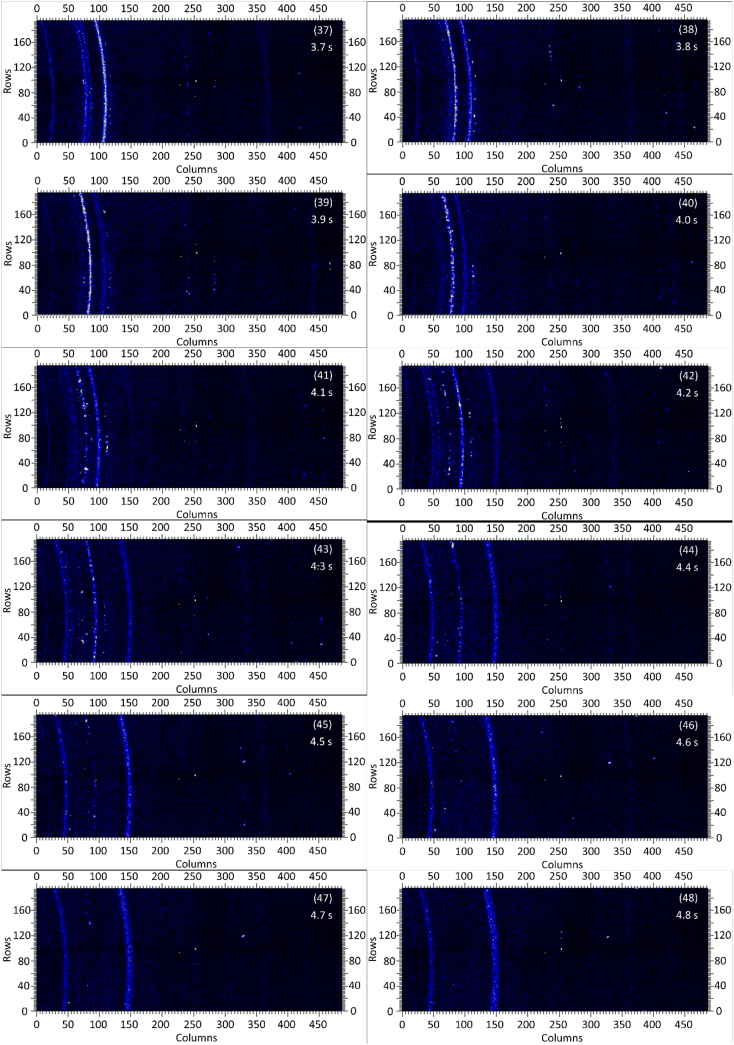

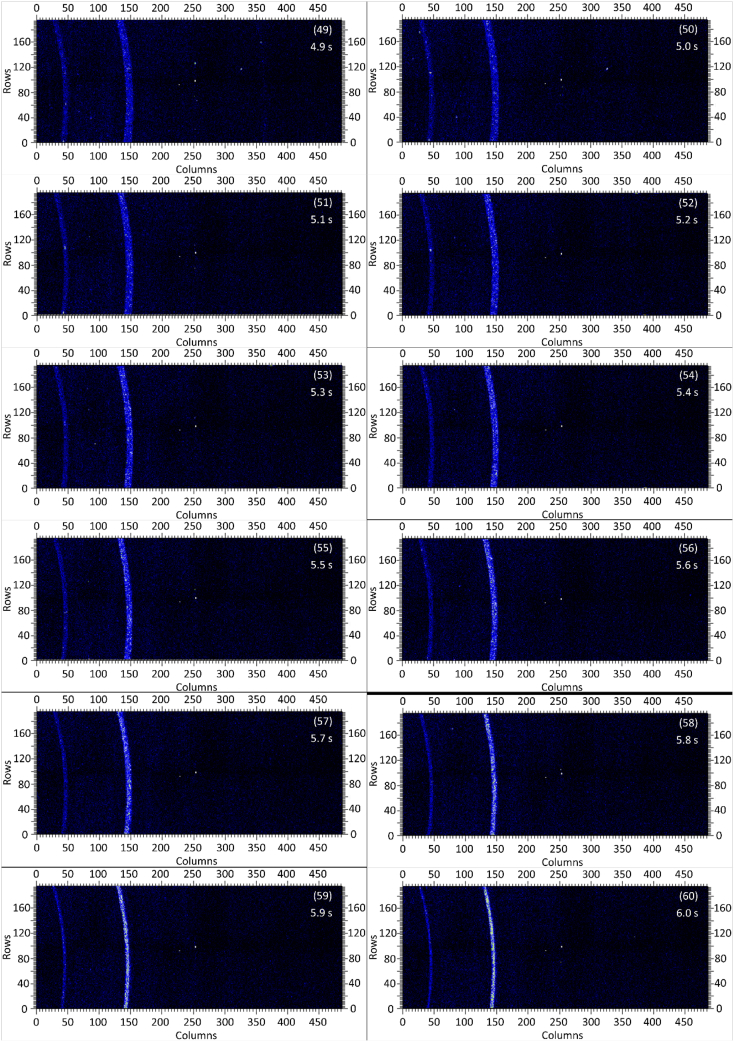

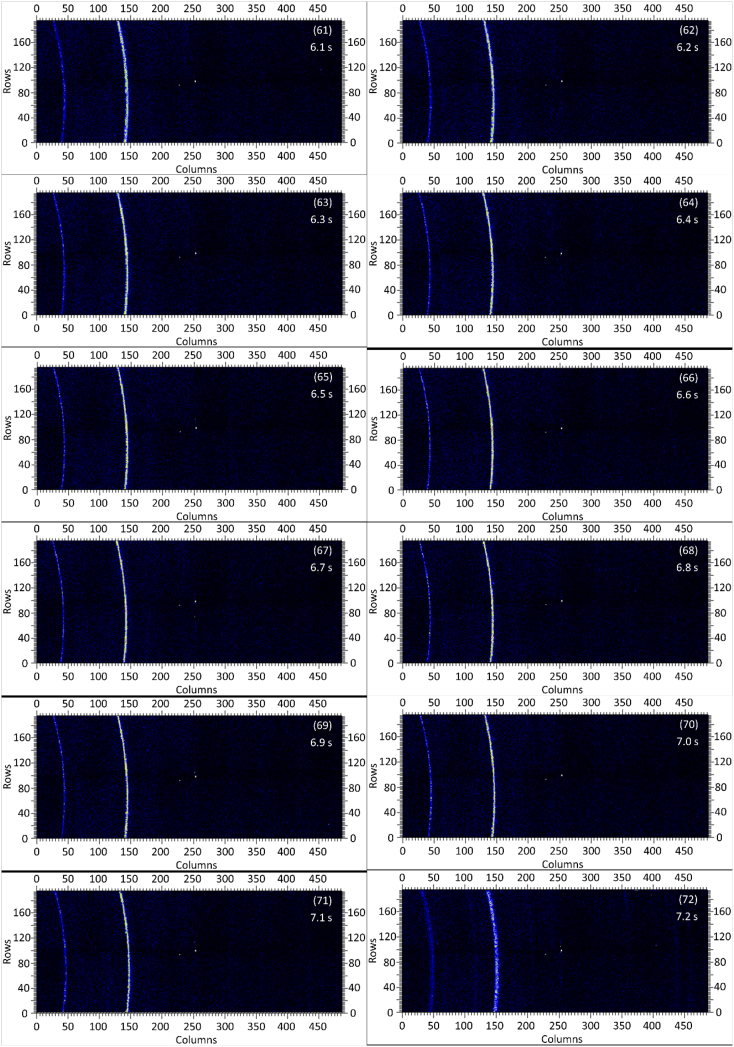

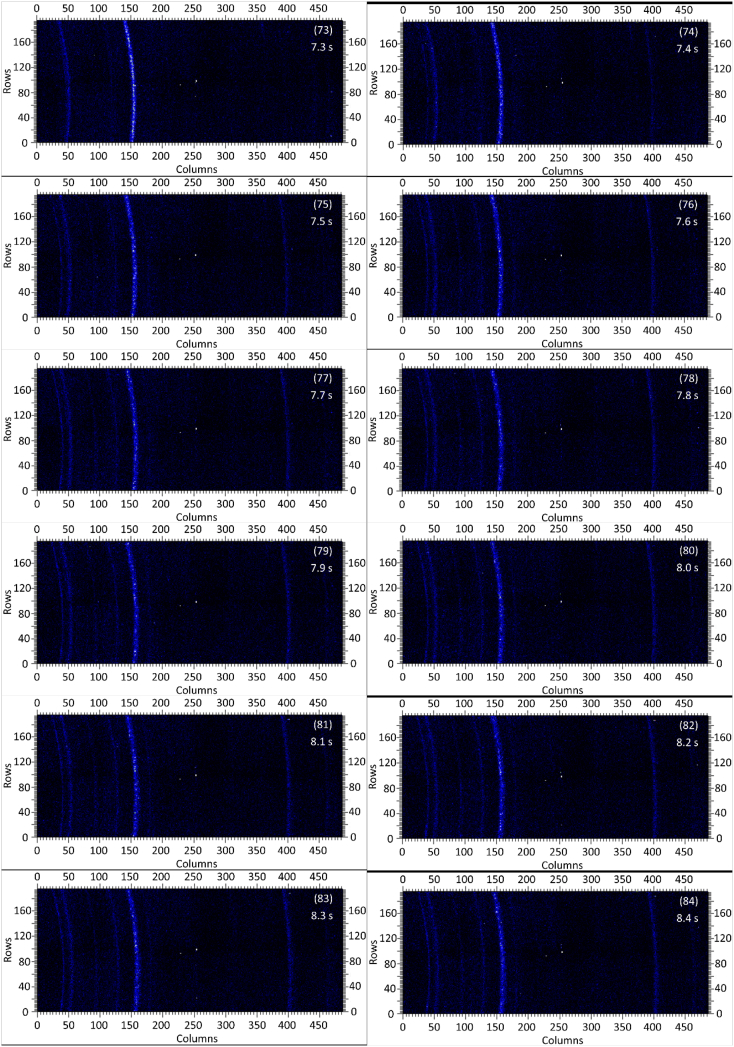

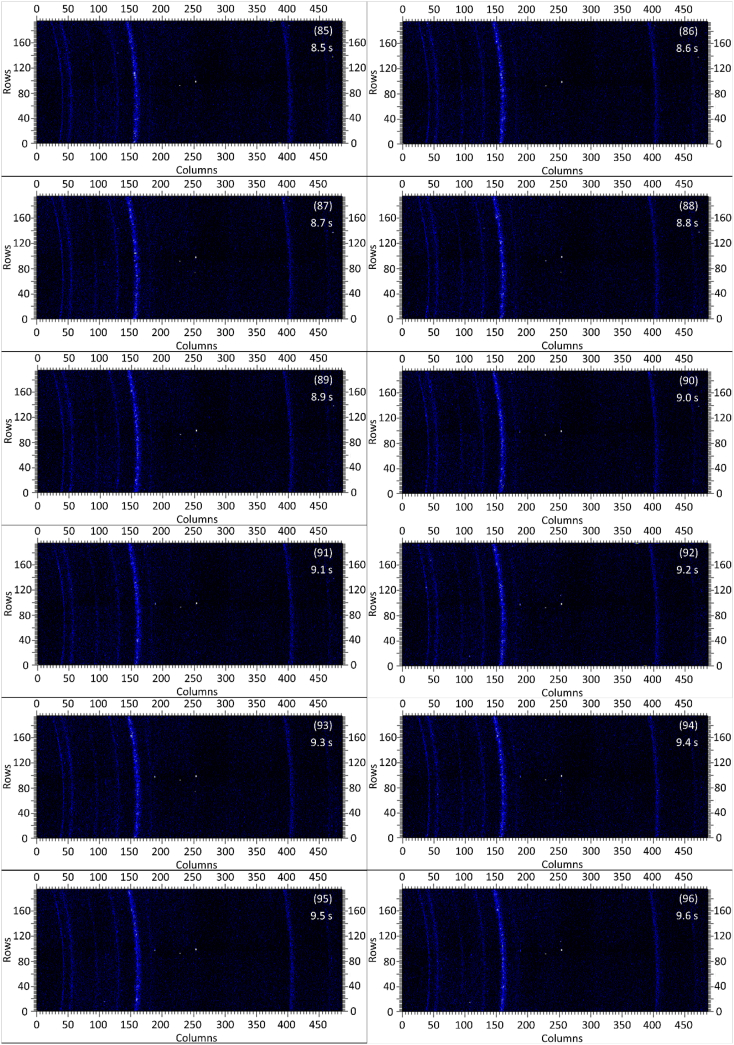

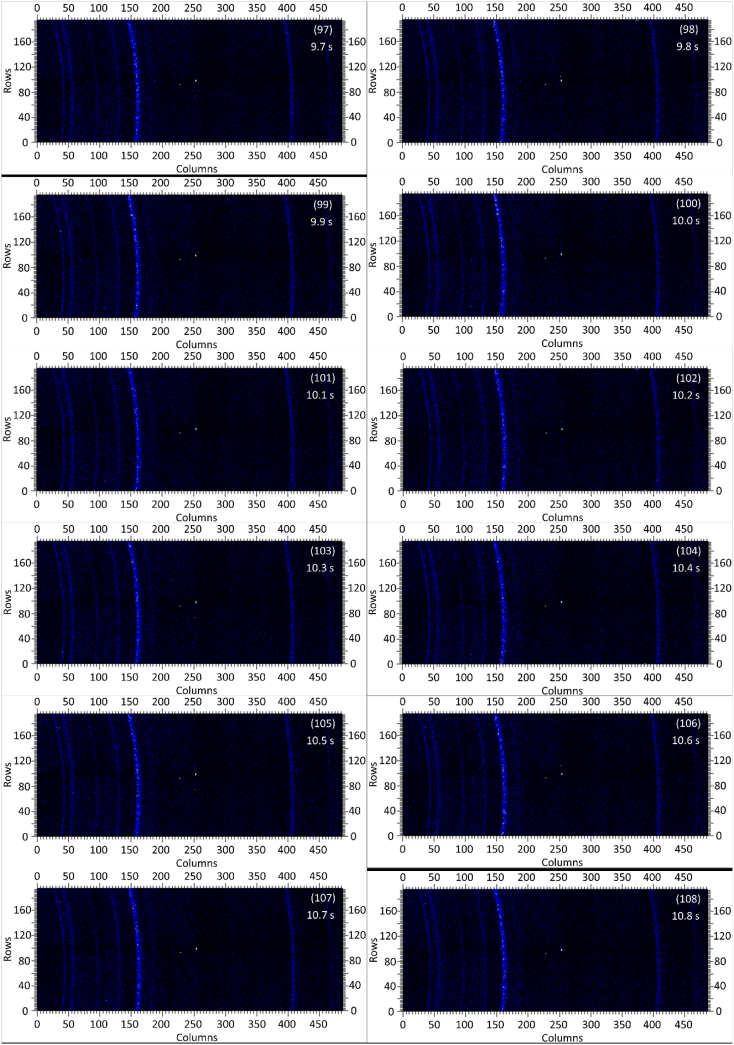

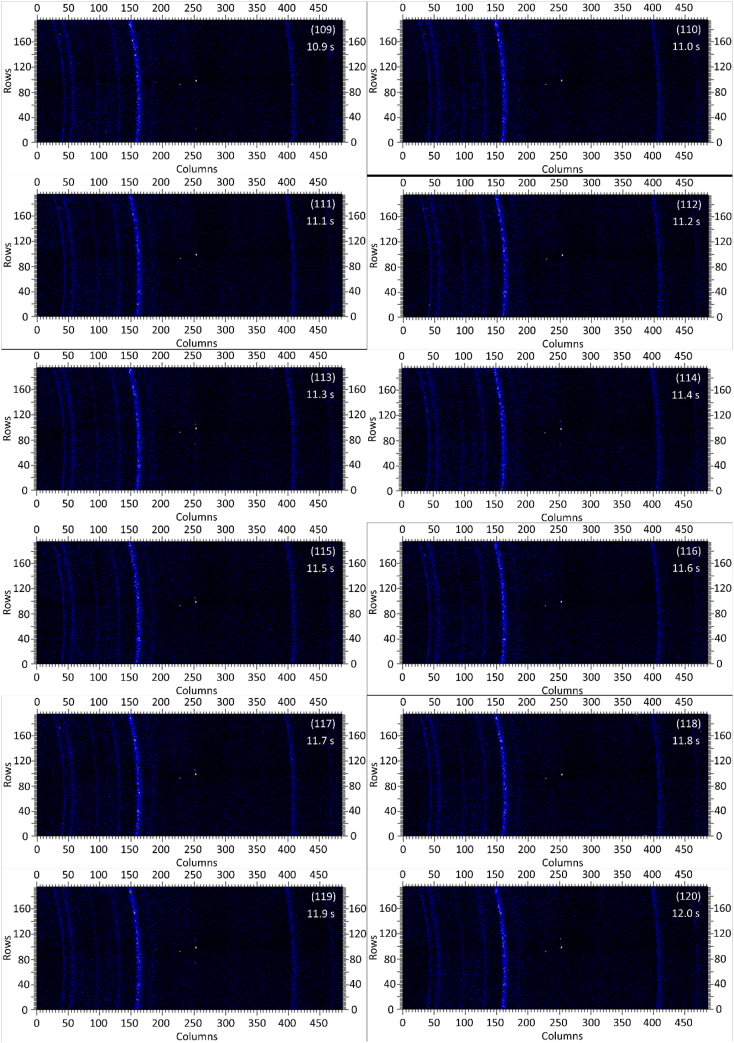
Image data (processed with FIT2D program) showing the variation of *in-situ* synchrotron X-ray diffraction patterns on the detector as a function of testing time (marked on the top right of each image). Unit of the two axes is pixel. The detector is 487 pixels × 195 pixels, with each pixel size 0.172 mm × 0.172 mm, and the size of the detector is 83.8 mm × 33.5 mm.

**Fig. 2:**
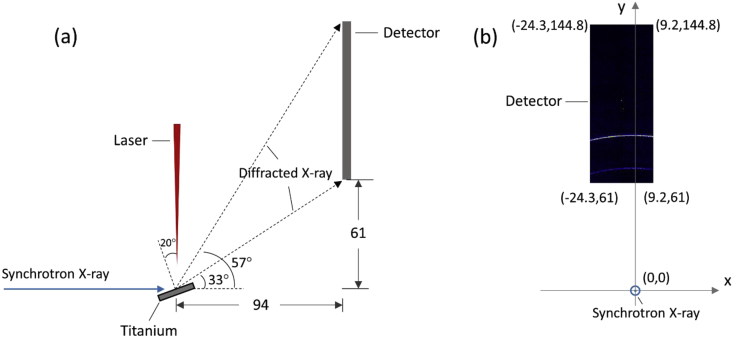
Images describing the positions of the parts of the in-situ synchrotron X-ray diffraction setup, and the unit for the length is millimeter (mm). (a) Parallel to the beam, (b) Normal to the beam.

The dataset in this article contains raw and preliminarily processed data obtained from the *in-situ* synchrotron X-ray diffraction test. With a data acquisition rate of 10 Hz and the testing time of 12 seconds, 120 individual data files in total were obtained. The raw sequencing data files (TIF file format) were deposited at Mendeley Database in the link aforementioned. The preliminarily processed data files (with FIT2D program) are shown in Fig. 1 (120 individual images), displaying the distribution of *in-situ* synchrotron X-ray diffraction patterns on the detector as a function of testing time. Fig. 2 shows the detailed information of the positions of every part of the *in-situ* X-ray diffraction setup.

## 2 Experimental design, materials, and methods

Pure titanium powders were used as the raw material (Alfa Aesar, 99.5 wt% purity, ∼325 mesh), and were first cold pressed into a disk (Ф25.4 × 2.5 mm^3^) using a stainless steel die and a pressing machine (MTI, type 16T), with the pressure 100 MPa and the dwelling time 60 seconds. A custom rig was designed for the *in-situ* synchrotron X-ray diffraction test, which comprised three main parts, namely the synchrotron X-ray beam, a laser heating source, and a 2D X-ray detector. The detailed descriptions can also be found in the authors’ previous work [1,2]. The synchrotron beam line (Protein Crystallography) located at the Center for Advanced Microstructures and Devices (CAMD), Baton Rouge, Louisiana, USA, was used, which had a 1.3 GeV electron storage ring, providing X-ray with a wavelength of 1.3808 Å and a beam energy around 8.9 keV. The synchrotron X-ray beam size was controlled with slits. To make sure the laser beam spot covering the X-ray beam spot, the X-ray beam was reduced to 0.3 mm × 0.3 mm. An IPG fiber laser (type YLS-2000, maximum power 2000 W, continuous wave mode, and Gaussian energy distribution) was utilized as the heating source. To record the X-ray diffraction data, a PILATUS 100 K detector system (Dectris AG, Switzerland, maximum framing rate 100 HZ) was applied.

The *in-situ* synchrotron X-ray diffraction obeys Bragg's equation, 2d⋅sinθ=nλ, where d, θ, n and λ represent interplanar spacing, half diffraction angle, positive integer and incident X-ray wavelength, respectively [3].

Prior to the test, the cold pressed titanium disk was attached to an aluminium sample holder with an adhesive bond to avoid slippage. The synchrotron X-ray beam was perpendicular to the laser beam direction. And the sample surface normal was inclined 20° to the laser beam direction (as shown in Fig. 2 (a)), to make sure an effective overlapping between the synchrotron X-ray and laser beam spots on the sample surface. The detector was rectangle shaped (83.8 mm × 33.5 mm), with the detector surface parallel to the laser beam direction (y axis in Fig. 2 (b)), and normal to the synchrotron X-ray beam direction (x axis). The sample to detector distance (in the direction of X-ray) was 94 mm, and the distance from the bottom of the detector to the X-ray beam was 61 mm. The X-ray beam was not strictly (also not necessarily) on the center axis of the detector, which is clearly indicated in Fig. 2 (b). The position of the bottom left corner of the detector was (−24.3,61)mm when considering the position of the X-ray beam as (0,0) on the same vertical plane. It is worth noting that the images in Fig. 1 were clockwise rotated 90° relative to the as-obtained diffraction data, with the long side oriented horizontally and the short side vertically. With such a rotation, the pattern orientates the same way with the regular X-ray diffraction data. The synchrotron X-ray beam and the detector (framing rate 10 Hz) were simultaneously turned on approximately 1 second before the initiation of laser, starting to record the diffraction data at room temperature. Around 1 second later, the laser beam was turned on to heat up the sample with a programmed profile. The laser power was first increased to 200 W with a constant rate of 40 W/s in 5 seconds, then kept at 200 W for 1 second, finally decreased to 0 W with a fixed rate of −200 W/s in 1 second. After the laser power was brought to 0 W, the X-ray beam and the detector were kept on working for about 4 more seconds. The whole testing process lasted for 12 seconds (120 frames recorded in total) and was under ambient air environment.
